# Aspartoacylase-LacZ Knockin Mice: An Engineered Model of Canavan Disease

**DOI:** 10.1371/journal.pone.0020336

**Published:** 2011-05-20

**Authors:** Nadine Mersmann, Dmitri Tkachev, Ruth Jelinek, Philipp Thomas Röth, Wiebke Möbius, Torben Ruhwedel, Sabine Rühle, Wolfgang Weber-Fahr, Alexander Sartorius, Matthias Klugmann

**Affiliations:** 1 Institute of Physiological Chemistry, University Medical Centre of the Johannes Gutenberg University, Mainz, Germany; 2 Department of Neurogenetics, Max-Planck-Institute of Experimental Medicine, Göttingen, Germany; 3 Neuroimaging Department, Central Institute of Mental Health, Mannheim, Mannheim, Germany; 4 Translational Neuroscience Facility, Department of Physiology, University of New South Wales, Sydney, New South Wales, Australia; Baylor College of Medicine, United States of America

## Abstract

Canavan Disease (CD) is a recessive leukodystrophy caused by loss of function mutations in the gene encoding aspartoacylase (ASPA), an oligodendrocyte-enriched enzyme that hydrolyses N-acetylaspartate (NAA) to acetate and aspartate. The neurological phenotypes of different rodent models of CD vary considerably. Here we report on a novel targeted aspa mouse mutant expressing the bacterial *β-Galactosidase* (*lacZ*) gene under the control of the *aspa* regulatory elements. X-Gal staining in known ASPA expression domains confirms the integrity of the modified locus in heterozygous aspa lacZ-knockin (*aspa^lacZ/+^*) mice. In addition, abundant ASPA expression was detected in Schwann cells. Homozygous (*aspa^lacZ/lacZ^*) mutants are ASPA-deficient, show CD-like histopathology and moderate neurological impairment with behavioural deficits that are more pronounced in *aspa^lacZ/lacZ^* males than females. Non-invasive ultrahigh field proton magnetic resonance spectroscopy revealed increased levels of NAA, myo-inositol and taurine in the *aspa^lacZ/lacZ^* brain. Spongy degeneration was prominent in hippocampus, thalamus, brain stem, and cerebellum, whereas white matter of optic nerve and corpus callosum was spared. Intracellular vacuolisation in astrocytes coincides with axonal swellings in cerebellum and brain stem of *aspa^lacZ/lacZ^* mutants indicating that astroglia may act as an osmolyte buffer in the aspa-deficient CNS. In summary, the *aspa^lacZ^* mouse is an accurate model of CD and an important tool to identify novel aspects of its complex pathology.

## Introduction

Aspartoacylase (ASPA) deacetylates N-acetyl-aspartate (NAA) to produce acetate and L-aspartate. This enzyme is a marker of mature oligodendrocytes [1], [2], [3] and mutations of the *aspa* gene cause the fatal recessive leukodystrophy Canavan disease (CD) [4], [5]. Patients suffer from mental retardation, hypotonia, seizures and death usually before the age of ten. Pathological changes include strongly elevated NAA levels in blood and urine, oligodendrocyte death and a progressive CNS vacuolization [6]. The underlying mechanisms of these multifaceted abnormalities are not understood and it is not clear to what extent the deficiency of ASPA in cell types other than oligodendroglia contributes to the development of clinical signs observed in CD. The monogenic nature of CD, and the lack of an effective treatment have provided the rationale for *in vivo* gene transfer into the CNS of patients and ASPA-deficient animals [7]. While these animal models generally reprise the pathological hallmarks observed in CD, they show substantial differences in disease severity and longevity [8], [9], [10]. The vast majority of clinical cases can be assigned to the relatively moderate infantile form of CD suggesting that an accurate animal model is expected to display a mild disease severity. Moreover, the identification of all ASPA expression domains is essential to gain a comprehensive picture of the complex CD pathology and design effective therapies.

Here, we describe an engineered mouse line expressing the bacterial *lacZ* gene under the control of the *aspa* promoter. Homozygous lacZ-knockin (*aspa^lacZ/lacZ^*) mutants are ASPA-deficient and show distinct abnormalities reminiscent of CD.

## Results

### Disruption of ASPA expression in aspa-lacZ knockin mice

**Figure 1:**
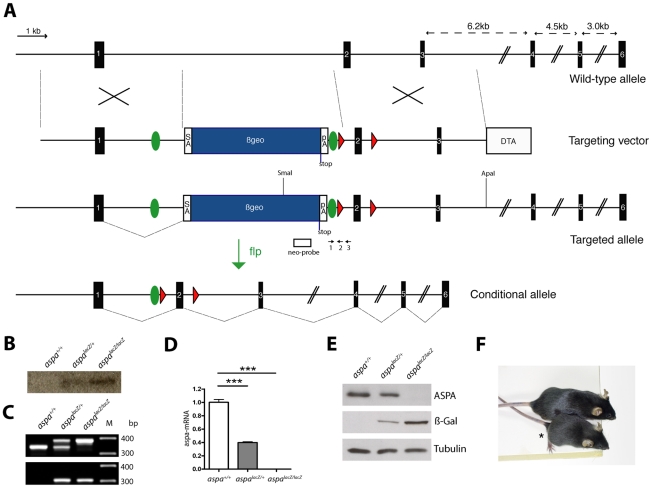
Generation of *aspa^lacZ^* mice by homologous recombination. **A,** Genomic structure of the murine *aspa* locus, which spans 6 exons (black boxes). Homologous recombination of the targeting vector inserts the βgeo cassette (blue box), encoding β-Galactosidase (lacZ), into intron 1 of the intact *aspa* gene. This cassette is flanked by frt-sites (green circles). Additional loxP sites flank exon 2 (red triangles). The vector includes a DTA cassette for negative selection. In the targeted allele, exon 1 is spliced to the splice acceptor site preceding βgeo, and transcription is terminated at the introduced pA site. The conditional *aspa^flox^* allele is produced by breeding *aspa^lacZ^* mutants to FLP-deleter mice [40] for recombination of the βgeo cassette *in vivo*. Note that frt and loxP sites are not drawn to scale. βgeo, β-galactosidase-neomycin resistance cassette; SA, splice acceptor; pA, polyadenylation site, DTA, Diphteria toxin gene. **B,** For Southern blot analysis genomic DNA was digested with SmaI/ApaI. The neo probe (white box in A) detected the expected 7.5 kb band in heterozygotes and homozygotes. **C,** Genomic PCR of littermates with primers 1 & 3 produces the expected amplicons in *aspa^+/+^* (336 bp), *aspa^lacZ/+^* (387 bp and 336 bp) and *aspa^lacZ/lacZ^* (387 bp) animals. The upstream loxP site was detected by PCR with primers 1 & 2 yielding a 307 bp band. **D,** Q-RT-PCR using a TaqMan probe for quantification of aspa mRNA levels in brains of *aspa^+/+^*, *aspa^lacZ/+^*, and *aspa^lacZ/lacZ^* mice (n = 3) confirms the attenuation of transcription downstream of exon 2 in the targeted allele. **E,** Representative Western blot of whole brain lysates of a*spa* ^+/+^, *aspa^lacZ/+^* and *aspa^lacZ/lacZ^* mice (aged 4 months, n = 3). The 37 kD protein ASPA was detected in *aspa* ^+/+^ and *aspa^lacZ/+^* mice but not in *aspa^lacZ/lacZ^* mutants. β-Galactosidase is expressed in *aspa^lacZ/+^* and *aspa^lacZ/lacZ^* brain but not controls. α-Tubulin was used as loading control. **F.** Representative picture of a male *aspa^lacZ/lacZ^* mutant (asterisk) and an *aspa^+/+^* littermate at P70.

Based on a knockout-first strategy that produces a knockout at the RNA processing level [11], targeting of the aspa locus in C57bl/6 ES cell was achieved by inserting the βgeo cassette (including the genes encoding β-Galactosidase and Neomycin) flanked by frt sites into intron 1 of the intact *aspa* gene. Additionally, exon 2 was flanked by loxP sites for optional conditional deletion of the targeted locus (Fig. 1A). The successful selection process after transfection of the targeting vector requires activity of the *aspa* gene locus in ES cells suggesting *aspa* expression at very early stages during development. Two clones with homologous recombination events were injected into blastocysts and germ line transmission confirmed after appropriate matings. Heterozygous *aspa^lacZ/+^* males and females were crossed to obtain *aspa^+/+^*, *aspa^lacZ/+^* and *aspa^lacZ/lacZ^* littermates at the expected Mendelian ratios (24.9%, n = 59; 51.9%, n = 123; 23.2%, n = 55) for the three genotypes. A single targeting event was confirmed by Southern blot analysis of genomic DNA obtained from liver of all three genotypes using a neomycin probe (Fig. 1B). The modified aspa locus could also be identified by PCR producing amplicons of the expected sizes (Fig. 1C). The βgeo cassette was flanked by an upstream 3’ splice acceptor site and a downstream transcriptional termination sequence resulting in an exon1-βgeo fusion transcript. The corresponding fusion protein contains the N-terminal 77 amino acid residues of ASPA with no predicted enzymatic activity [12]. Because inactivation of the *aspa* gene was designed to attenuate transcription upstream of exon 2, we determined aspa mRNA levels in the brain of *aspa^+/+^*, *aspa^lacZ/+^* and *aspa^lacZ/lacZ^* mice (n = 3) by quantitative real-time (Q-RT) PCR. Using a TaqMan probe binding to a downstream sequence, aspa mRNA levels were undetectable in *aspa^lacZ/lacZ^* mice, while we observed a 60.4±4.3% (p = 0.0001) reduction of mRNA expression in *aspa^lacZ/+^* brains compared to controls (Fig. 1D). We then used a rabbit anti-ASPA antibody to determine the ASPA protein levels in immunoblot analysis of whole brain homogenates of 2 months old *aspa^+/+^*, *aspa^lacZ/+^* and *aspa^lacZ/lacZ^* mice (n = 3). In the presence of one functional allele in heterozygous *aspa^lacZ/+^* animals, ASPA was reduced to 74.5±5.7% (p = 0.048) of control levels and was completely abolished in homozygous *aspa^lacZ/lacZ^* mutants (Fig. 1E). Analysis of β-Galactosidase levels showed the complementary profile with moderate levels in *aspa^lacZ/+^* mice and increased levels in *aspa^lacZ/lacZ^* mutants reflecting gene dosage effects. Phenotypically, *aspa^lacZ/lacZ^* mutants were smaller (Fig. 1F) and showed gait abnormalities starting before weaning. The body weight of *aspa^lacZ/lacZ^* mice was significantly decreased at P40 compared with controls for both sexes (*aspa^+/+^* 19.05±0.83 g vs. *aspa^lacZ/lacZ^* 14.70±0.67 g males, *p<0.001*; *aspa^+/+^* 16.84±0.30 g vs. *aspa^lacZ/lacZ^* 13.93±0.58 g females, *p<0.001*). Using flp-deleter mice, the *aspa^lacZ/lacZ^* allele could be converted via excision of the βgeo cassette to the floxed *aspa^f/f^* allele (Fig. 1A). The latter is phenotypically identical to the wildtype allele and will be instrumental for conditional deletion of the *aspa* gene via Cre-mediated recombination (our unpublished observations).

### ASPA expression domains

**Figure 2:**
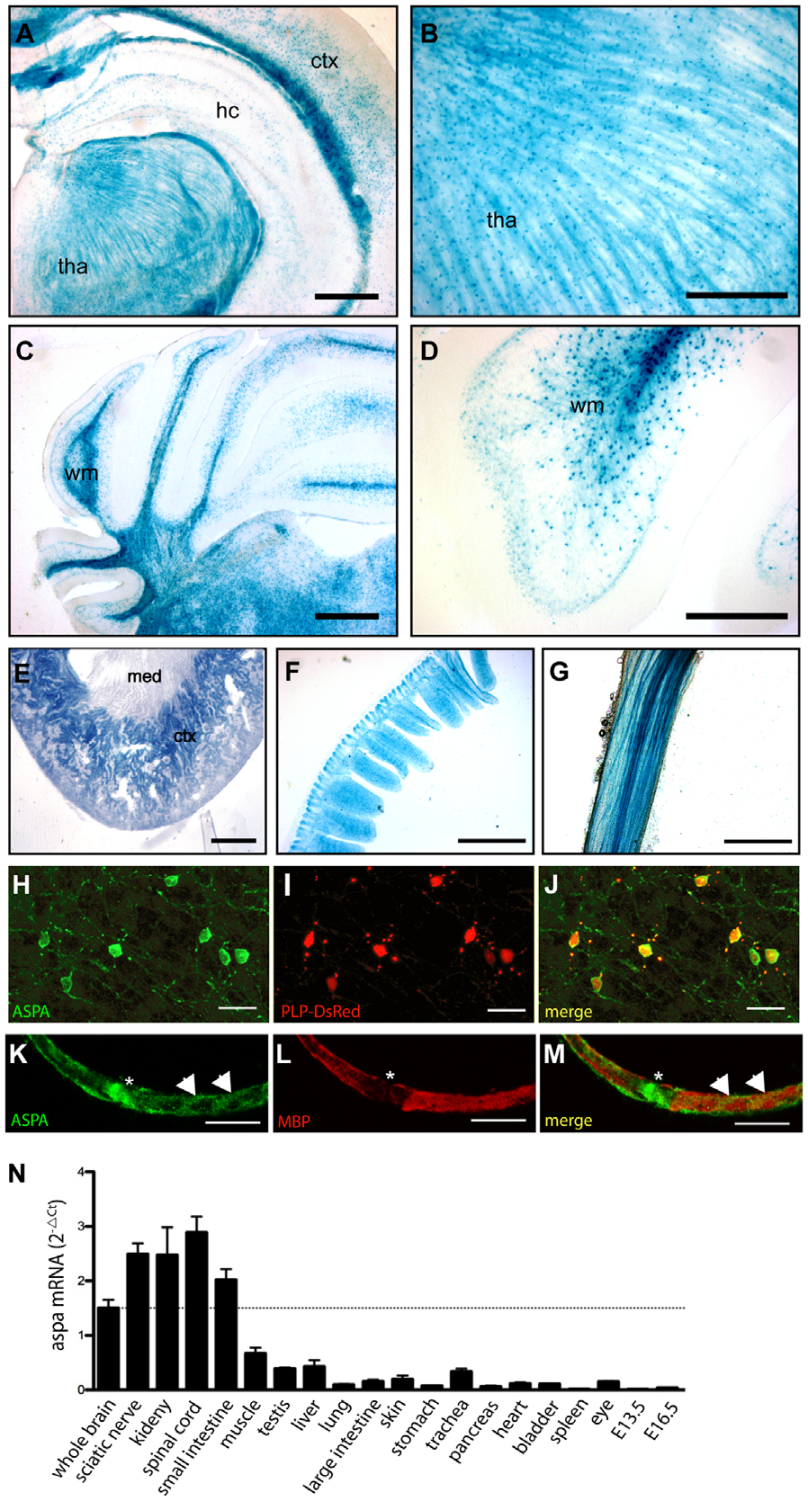
Analysis of *aspa* expression in CNS and periphery. **A–D,** Representative pictures of β-Galactosidase staining in adult *aspa^lacZ/+^* mouse tissues show activity of the *aspa* gene in oligodendrocytes of white and grey matter throughout the brain. Abundant staining was detected in white matter tracts in cerebellum and corpus callosum. While in the hippocampus and cortex X-Gal staining was found in oligodendrocyte cell bodies (A), lacZ-positive fibres were detected in the thalamus (B). In the cerebellum, proximal processes and somata of white matter oligodendrocytes were stained (C, D). **E**–**F,** Outside the CNS, the cortex of the kidney (E), small intestine (F) and sciatic nerve fibres (G) show intense X-Gal staining. **H**–**J,** Confocal detection of ASPA immunoreactivity (H) in cerebellar white matter of an adult Plp-DsRed-1 transgenic mouse expressing the red fluorescent reporter protein (I) in oligodendrocytes [15]. ASPA is expressed in somata and processes of oligodendrocytes. The pattern of ASPA-immunopositive cells matches the DsRed-expressing oligodendrocytes (J). **K**–**M,** Confocal detection of ASPA immunoreactivity in cytosolic domains of wildtype Schwann cells. ASPA is enriched at the paranode (asterisk) and bands of Cajal (arrow heads) and segregates from MBP. **N,** Q-PCR analysis of aspa mRNA levels in different tissues of adult *aspa^+/+^* mice (n = 6). hc, hippocampus; ctx, cortex; med, medulla; tha, thalamus; wm, white matter. Bars: 500 µm in A,C,E; 200 µm in B, D; 400 µm in F,G; 20 µm in H–J; 10 µm in K–M.

The genomic configuration of the modified locus was designed to recapitulate the activity of the aspa promoter and analysis of *aspa^lacZ/+^* heterozygots by X-Gal immunohistochemistry detected the presence of the soluble enzyme β-Galactosidase in central and peripheral tissues (Fig. 2 A–G). X-Gal was detected in oligodendrocytes throughout the brain. Intense staining was observed in corpus callosum, but was also present in subcortical grey matter (Fig. 2A). Fibre tracts in the thalamus showed strong X-Gal staining indicating the presence of β-Galactosidase in oligodendrocyte processes (Fig. 2B). Brain stem and cerebellar white matter also displayed abundant X-Gal signals (Fig. 2C). At higher magnification, X-gal signal was visualized in the soma and proximal processes of oligodendrocyte in the cerebellum (Fig. 2D). We also investigated the activity of the aspa promoter in the periphery and observed abundant X-Gal staining in small intestine and kidney (Fig. 2E,F) confirming previous reports on ASPA expression in these organs [7], [13], [14]. X-Gal staining in the kidney was found in proximal tubules, but was absent from medulla and glomeruli. This expression pattern matches the distribution of ASPA immunoreactivity in this organ [14]. We also observed X-Gal staining in fibres of the sciatic nerve (Fig. 2G). However, the histological approach did not resolve whether β-Galactosidase was localized to Schwann cells or the underlying axons. No unspecific staining was observed in identically processed tissues from *aspa^+/+^* controls (not shown).

We then determined ASPA immunoreactivity specifically in oligodendroctyes of homozygous Plp-DsRed-1 transgenic mice expressing the RFP reporter protein under the control of the plp-1 promoter [15]. On the subcellular level, staining was detected in soma and proximal processes of oligodendrocytes (Fig. 2H–J) confirming previous findings by us and others [2], [3]. Double immunohistochemical detection revealed that immunoreactivities of both β-Galactosidase and ASPA exactly co-localized in the brain of heterozygous *aspa^lacZ/+^* mice further validating this reporter line as a genetic tool to monitor the activity of the aspa gene (Supporting Information Figure S1).

To discern ASPA expression in the sciatic nerve, we performed confocal analysis of ASPA immunoreactivity with teased nerve fibres of *aspa^+/+^* mice (Fig. 2K–M). ASPA was detected in protoplasmic domains of Schwann cells and segregated from the myelin marker MBP. Our results show that ASPA is present in myelinating glia of the central and peripheral nervous system. Since the histological approach precludes quantitative analyses we conducted Q-PCR to determine the relative abundances of aspa mRNA in different organs obtained from adult *aspa^+/+^* animals. Expression levels in kidney, spinal cord, small intestine, and sciatic nerve were almost two-fold more than that in brain (Fig. 2N). Since peripheral neurons were devoid of ASPA immunoreactivity (Fig. 2K–M), our PCR data suggest that Schwann cells are the main source of aspa mRNA in the sciatic nerve. Moderate levels of expression were detected in muscle, testis and lung, and negligible in whole body preparation at embryonic day E13.5 and E16.5.

### CD-like histopathology in *aspa^lacZ/lacZ^* mutants

**Figure 3:**
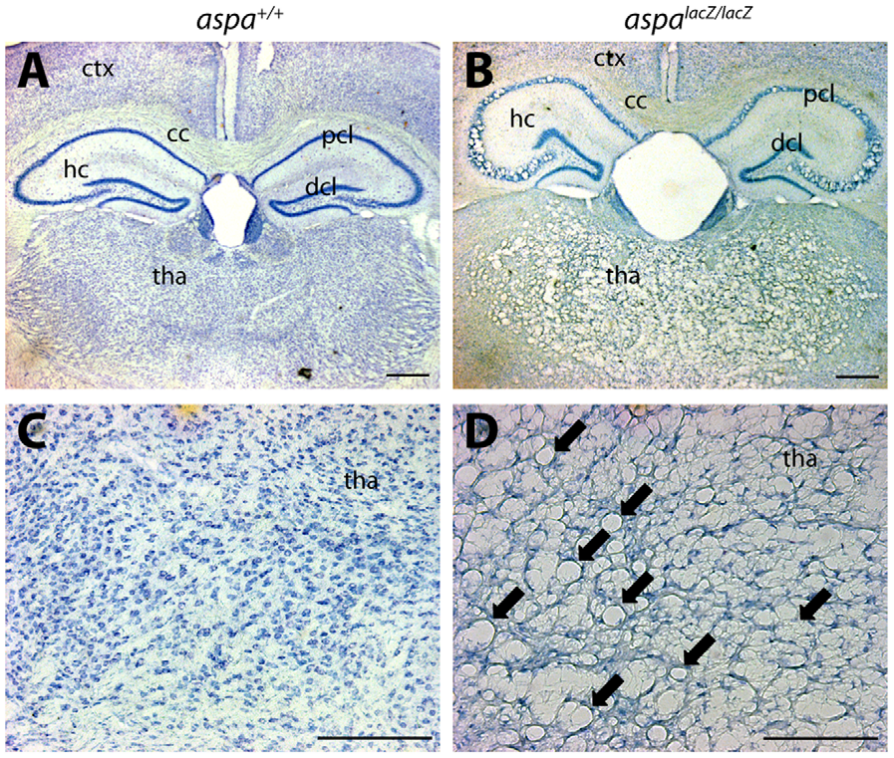
CNS vacuolisation in *aspa^lacZ/lacZ^* mutants. Representative pictures of Nissl stained brain sections of *aspa^+/+^* (A,C) and *aspa^lacZ/lacZ^* (B,D) mice (4 months). Vacuoles are abundant in forebrain grey matter of cortex and thalamus while white matter of the corpus callosum is spared (B). In the hippocampus, the histopathology is exclusively seen in the pyramidal but not the dentate granule cell layer. Magnifications of thalamic areas show substantial spongy degeneration in the mutant (arrows in D) but not in the control (C). hc, hippocampus; cc, corpus callosum; ctx, cortex; dcl, granule cell layer; pcl, pyramidal cell layer; tha, thalamus. Bars: 400 µm in A,B; 200 µm in C,D.

**Figure 4:**
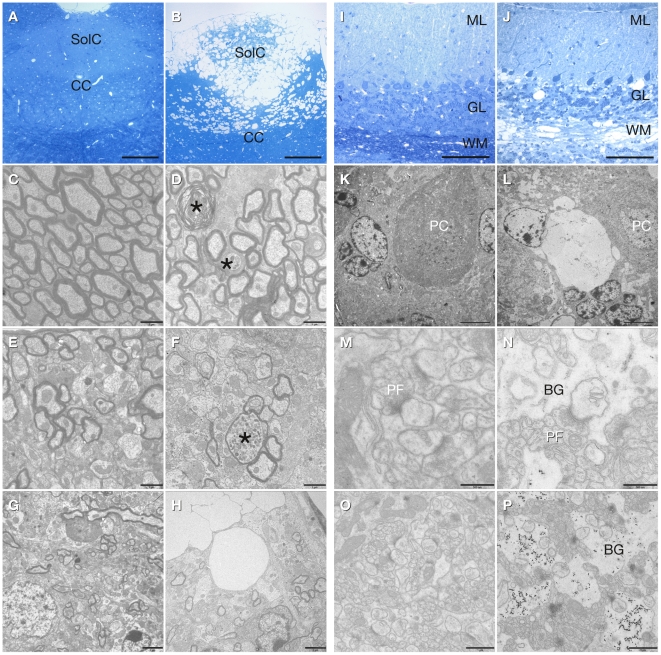
Histopathology of the brain stem and cerebellum in *aspa^lacZ/lacZ^* mice. Caudal brain stem of controls (**A, C, F, G**) and *aspa^lacZ/lacZ^* mice (**B, D, E, H**): Vacuolization is abundant in the dorsal region *aspa^lacZ/lacZ^* mice (solitary tract nu, commissural, SolC) as shown in semithin sections (**B**) and electron microscopy (**H**). In the pyramidal tract of mutant mice myelin appears normal, but axonal swellings and degeneration are present (**D**, asterisks). Axonal swellings as well as hypomyelination were found in the dorsal region with abundant vacuolization (**F**, asterisk). Cerebellum of controls (**I, K, M, O**) and *aspa^lacZ/lacZ^* mice (**J, L, N, P**): In semithin sections of mutant cerebellum, vacuoles are detectable in the white matter (WM), granule cell layer (GL) and in the Purkinje cell layer (**J, L**). Parallel fibre to Purkinje cell dendrite synapses appear normal (**M, N**), but in *aspa^lacZ/lacZ^* mice the Bergman glia (BG) is swollen and shows accumulation of electron dense particles (**N, P**). BG, Bergman glia; CC, central canal; GL, granule cell layer; ML, molecular layer; PC, Purkinje cell; PF, parallel fibre; SolC, solitary tract nu, commissural; WM, white matter. Scale bars: 100 µm (**A, B, I, J**), 5 µm (**K, L**), 2 µm (**G, H**), 1 µm (**C, D, O, P**), 500 nm (**M, N**).

Histological analysis of Nissl stained brain sections of *aspa^lacZ/lacZ^* mutants (4 months old) revealed spongy degeneration throughout the CNS. Vacuolisation was prominent in hippocampus, thalamus, brainstem, cerebellum, and spinal cord. In contrast, the white matter tracts of corpus callosum and optic nerve were remarkably intact (Supporting Information Figure S2). In the hippocampus, vacuoles occurred specifically in the pyramidal cell layer while dentate granule cells were spared (Fig. 3A and B). In contrast, the thalamus was uniformly affected (Fig. 3C and D). Spongy degeneration was abundant in the dorsal region of the brain stem (Fig. 4A, B, G, H). EM analysis revealed swollen and degenerated axons in the presence of normal myelin in the ventral aspect of the brain stem (Fig. 4C, D). In the dorsal brain stem hypomyelination and axonal swellings were observed (Fig. 4E, F). In the cerebellum, vacuolisation was detected in white matter, the granule cell layer and the Purkinje cell layer but not in the molecular layer (Fig 4I, J, K, L). At the ultrastructural level, swollen Bergman glia showed accumulations of electron dense particles (Fig. 4N, P). The nature of the latter could not be determined. Interestingly, the parallel fibre to Purkinje cell dendrite synapses appeared normal (Fig. 4M, N).

### Regulation of myelin associated proteins

**Figure 5:**
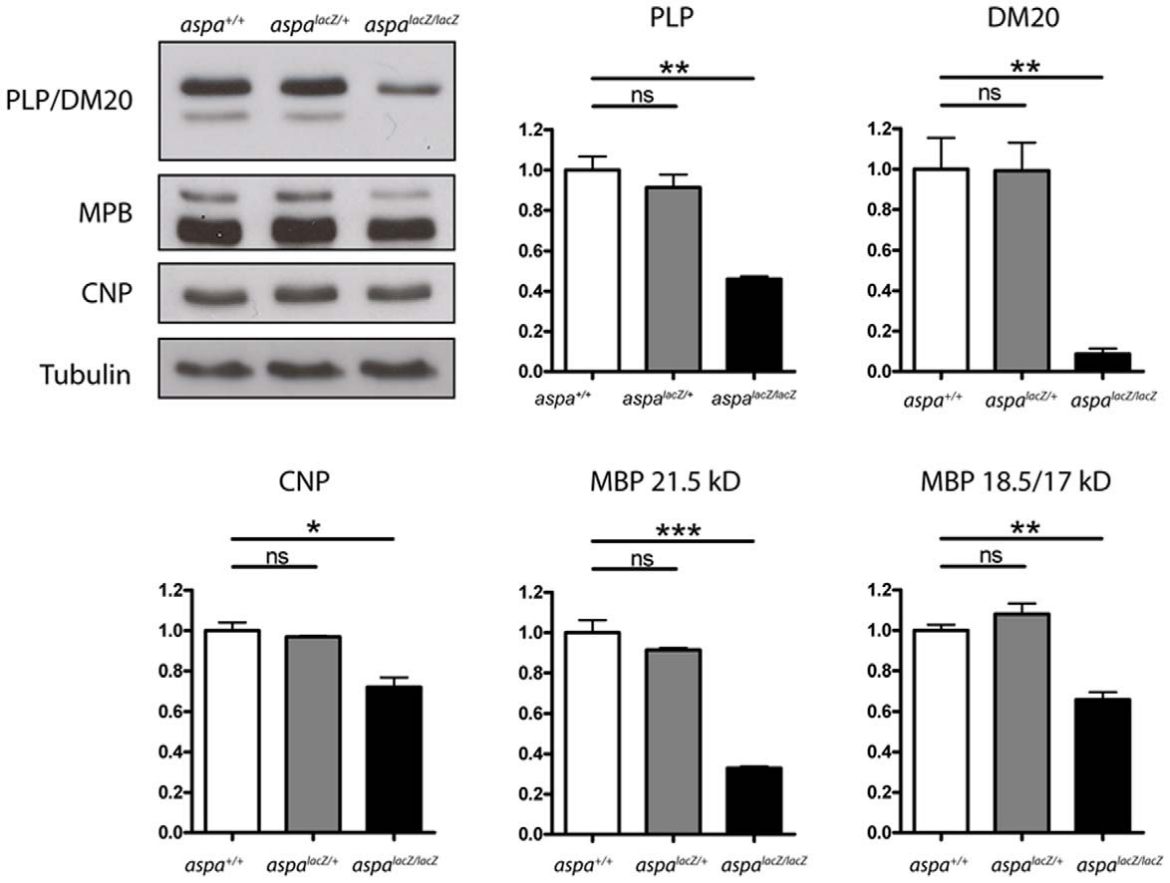
Myelin protein levels are reduced in *aspa^lacZ/lacZ^* mutants. Western blot analysis of PLP, DM20, CNP and MBP levels in whole brain lysates of *aspa^+/+^*, *aspa^lacZ/+^* and *aspa^lacZ/lacZ^* mice (P60, n = 3). Quantification of immunoblots shows statistically significant decreases of all proteins tested in homozygous mutants while the presence of one functional aspa allele is sufficient to preserve protein quantities at control levels. Note: PLP, DM20, and the MBP21.5 kD and MBP18.5/17 kD isoforms were analysed separately.

Levels of myelin proteins were investigated by immunoblot analysis of whole brain lysates of *aspa^+/+^*, *aspa^lacZ/+^* and *aspa^lacZ/lacZ^* littermates (at 2 months; n = 3 per group). In homozygous mutants, the major CNS myelin protein PLP and its smaller isoform, DM20, were reduced to 45.9±1.4% (p = 0.001) and 8.7±2.7% (p = 0.004), respectively (Fig. 5). The reason for the relatively stronger regulation of DM20 is unclear. The 21.5 kD and 18.5/17 kD MBP isoforms were reduced to 32.9±0.9% (p<0.001) and 65.7±3.8% (p = 0.002), respectively. CNP was only moderately downregulated to 72.0±4.8% (p = 0.011). Heterozygous *aspa^lacZ/+^* mice did not differ from *aspa^+/+^* controls. These results further suggest that demyelination is present in *aspa^lacZ/lacZ^* mutants.

### Reactive gliosis in *aspa^lacZ/lacZ^* mutants

**Figure 6:**
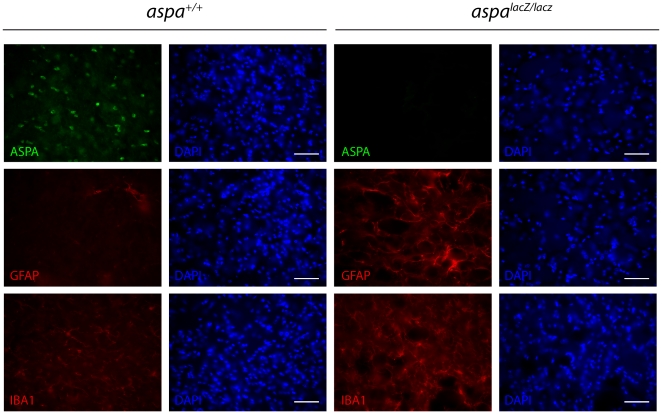
Reactive gliosis in *aspa^lacZ/lacZ^* mice. Immunohistochemical staining in the thalamus of *aspa^+/+^* controls and *aspa^lacZ/lacZ^* mice showed substantially increased numbers of GFAP-positive astrocytes and Iba-1-positive microglia in the mutants (at 3 months of age). Note that ASPA immunoreactivity is present in the control but not in the mutant. Bars: 25 µm.

Increased numbers of astrocytes and microglia have been reported in CD [16]. Therefore, we performed immunohistochemical analyses using brain sections of controls and mutants characterized by the presence (*aspa^+/+^*) or absence (*aspa^lacZ/lacZ^*) of ASPA immunoreactivity (Fig. 6). Microglia activation in the mutant thalamus was detected by Iba-1 immunohistochemistry (Fig. 6). Assessment of glial acidic fibrillary protein (GFAP) immunoreactivity revealed increased astrocyte numbers in the thalamus of *aspa^lacZ/lacZ^* mice. Astrogliosis was less prominent in white matter tracts where vacuolisation did not occur, such as corpus callosum (not shown).

### Metabolic imbalances in *aspa^lacZ/lacZ^* mutants

**Figure 7:**
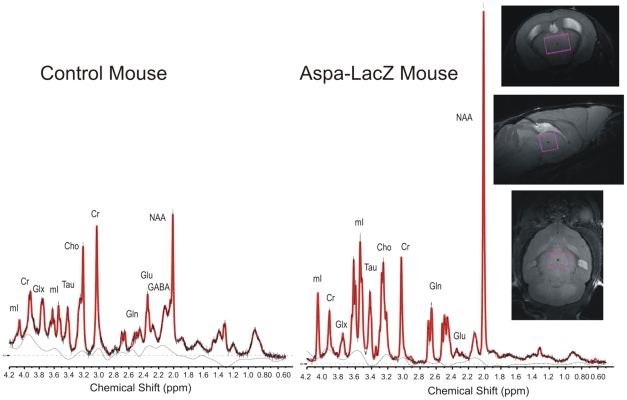
^1^H-MRS analysis. Spectra were acquired from *aspa^+/+^* and *aspa^lacZ/lacZ^* littermates using a 2×2×3 mm^3^ single-voxel PRESS Sequence in the thalamus. Representativ metabolites are marked in the spectra (Cr: Creatine and Phosphocreatine, NAA: NAA+NAAG, Cho: Phosphocholine and Glycerophosphorylcholine, Glu: Glutamate, Gln: Glutamine, mI: Myo-Inositol, Tau: Taurine, Glx: Glu+Gln). For metabolite concentrations see Table 1.

The lack of ASPA leads to accumulation of its substrate NAA in brains of patients and CD models [7], [10]. Ultrahigh field proton magnetic resonance spectroscopy (^1^H MRS) at 9.4T allows non-invasive quantification of metabolites, like NAA, glutamate, glutamine, GABA, taurine, and glucose in the living rodent brain (Fig. 7). We focused on the thalamus and prefrontal cortex because these regions showed high and low degree of vacuolization, respectively. We found substantially more of NAA (255.8%), myo-inositol (330.6%), taurin (204.4%) and glutamin (122.0%) in the *aspa^lacZ/lacZ^* thalamus compared to the control, while glutamate was reduced by 67% and GABA by 31.4% (Table 1). Due to limitations in resolution, the N-acetylaspartylglutamate (NAAG) peak could not be discerned from the NAA signal. While in the prefrontal cortex there was an increase in NAA (75.5%), myo-inositole (75.4%) and taurine (22.4%), these changes were not as prominent as in the thalamus. Of note, the reduction in GABA (26.4%) was similar to that seen in thalamus while creatine was virtually unchanged in both areas.

**Table 1:** Metabolite concentrations in the *in vivo* mouse brain measured with ^1^H-MRS over a 2*2*3 mm^3^ and 3*1.2*2.8 mm^3^ volume of interest in the thalamic region and the prefrontal cortex, respectively.

	Thalamus							Prefrontal Cortex						
	Control mouse[mM/l] ± %SD			Aspa-LacZ[mM/l] ± %SD			Change%	Control mouse[mM/l] ± %SD			Aspa-LacZ[mM/l] ± %SD			Change%
GABA	3.84	±	4	2.63	±	10	−31.4%	2.65	±	7	1.95	±	22	−26.4%
Glc	6.19	±	6	4.63	±	12	−25.2%	7.19	±	5	N/A	±	N/A	N/A
Tau	5.50	±	3	16.75	±	2	204.4%	11.53	±	2	14.12	±	3	22.4%
Gln	3.65	±	4	8.12	±	3	122.2%	2.36	±	6	2.38	±	14	0.7%
Glu	9.28	±	2	3.06	±	9	−67.0%	11.21	±	2	8.25	±	5	−26.4%
mI	8.06	±	2	34.70	±	1	330.6%	6.59	±	3	11.56	±	5	75.4%
Lac	5.08	±	4	2.55	±	12	−49.9%	3.90	±	6	3.48	±	16	−10.9%
Ch	2.58	±	2	1.30	±	4	−49.5%	2.35	±	2	2.59	±	5	10.4%
NAA	11.56	±	2	41.13	±	1	255.8%	11.21	±	2	19.68	±	3	75.5%
Cr	9.66	±	1	10.75	±	2	11.4%	9.50	±	2	9.50	±	4	0.0%

The standard deviation of the metabolite concentrations (SD) corresponds to the Cramér-Rao lower bounds of the LCModel-fit and is given in %. SD-values below 20% are considered reliable. Error bars are derived through the fit-routine and indicate the quality of individual metabolite measurements. Cr: Creatine and Phosphocreatine, NAA: NAA+NAAG, Cho: Phosphocholine and Glycerophosphorylcholine, Glu: Glutamate, Gln: Glutamine, mI: Myo-Inositol, Tau: Taurine, Glc: Glucose.

Interstitial NAA levels can also increase via the hydrolysis of NAAG by the astrocyte-specific plasma membrane enzyme folh1/glutamate carboxypeptidase II (EC 3.4.17.21). Therefore we investigated the mRNA expression levels of this enzyme and found no changes in *aspa^lacZ/+^* (103.9±6.9%; p = 0.652) but a reduction of 48.1±3.6% (p<0.0001) in *aspa^lacZ/lacZ^* mice (Supporting Information Figure S3). This decrease was not due to a reduction in astrocyte numbers (Fig. 6) and might represent a cellular mechanism to prevent additional generation of NAA from NAAG in an environment of pathologically enriched NAA.

### Sexual dimorphism in behavioural outputs of *aspa^lacZ/lacZ^* mice

**Figure 8:**
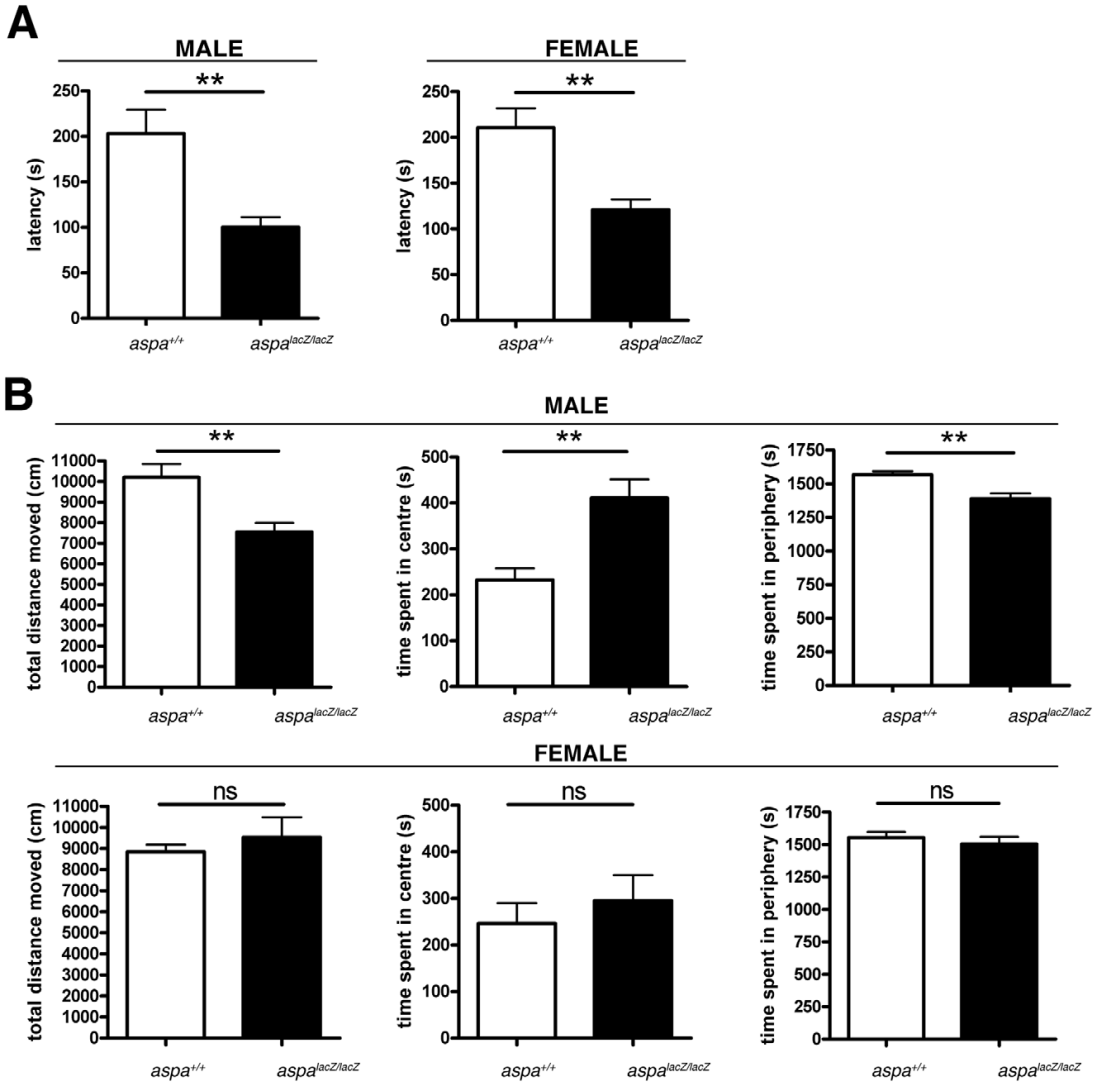
Special behavioural deficits in male but not female *aspa^lacZ/lacZ^* mutants. **A.** Analysis of motor coordination in the rotarod test shows impairment of both female and male *aspa^lacZ/lacZ^* mice compared with their *aspa^+/+^* controls at P90. **B.** Spontaneous exploratory activity and aspects of anxiety-related behaviour of both sexes and genotypes were tested in an open field for 30 min (see Methods section). Male mutants (n = 8) travelled less and spent more time in the centre than male controls (n = 8). This behaviour is suggestive of impaired exploratory activity and anxiolysis. The behaviour of female mutants (n = 6) did not differ from female controls (n = 7).

CD is characterized by general muscle weakness and the failure to develop motor control. We observed ataxia with splayed hind legs and a general lack of muscle tonus in male and female mutants but not their respective littermate controls starting at the age of 3–4 weeks. These gait abnormalities progressed gradually to obvious deficits in motor coordination, and weakness especially in the hind limbs. Supporting Video S1 shows representative behaviour of male mutant and control mice at 2 months of age. At the time of this report, all *aspa^lacZ/lacZ^* mutants had survived >1.5 years suggesting normal longevity. However, they showed progressive immobility and impaired motor function characterized by spasticity and jerky movements. When we started to monitor motor learning and general locomotion in specialised behavioural tests, we used aged-matched (P90) cohorts of mutant and wt mice that included pools of both sexes, and obtained inconsistent data. We then analysed male *aspa^lacZ/lacZ^* and *aspa^+/+^* littermates separately from the female cohorts. To quantify behavioural deficits in younger animals, we assessed their motor coordination using the rotarod test. At P90, we observed significant differences within both male (*aspa^+/+^* 203.3±26.2 s vs. *aspa^lacZ/lacZ^* 100.2±11.0 s; p = 0.0012; two-tailed t-test) and female (*aspa^+/+^* 210.5±21.2 s vs. *aspa^lacZ/lacZ^* 120.9±11.2 s; p = 0.0046) cohorts (Fig. 8A). We also investigated the inherent locomotor and exploratory activity of both cohorts in an open field (Fig. 8B). We found that motor activity monitored by the total distance travelled over a 30 min period, was reduced in male (*aspa^+/+^* 10206±642.1 s vs. *aspa^lacZ/lacZ^* 7547±439.2 s; p = 0.0042; two-tailed t-test) but not in female mutants compared with sex-matched controls (*aspa^+/+^* 8838±340.9 s vs. *aspa^lacZ/lacZ^* 9527±955.0 s; p = 0.4849; two-tailed t-test). Male mutants spent more time in the centre of the test arena than controls (*aspa^+/+^* 232.6±25.3 s vs. *aspa^lacZ/lacZ^* 411.2±40.1 s; p = 0.0071; two-tailed t-test) and only slightly less time in the periphery (*aspa^+/+^* 1567±25.3 s vs. *aspa^lacZ/lacZ^* 1389±40.1 s; p = 0.0021; two-tailed t-test), suggesting reduced anxiety-like behaviour. In contrast, there was no difference within the female cohort (Center: *aspa^+/+^* 246.5±44.0 s vs. *aspa^lacZ/lacZ^* 295.2±55.2 s; p = 0.4989; Periphery: *aspa^+/+^* 1554±44.0 s vs. *aspa^lacZ/lacZ^* 1505±55.2 s; p = 0.4989; two-tailed t-test).

## Discussion

We describe a novel ASPA-deficient mouse mutant obtained by a targeted insertion of the βgeo cassette in-frame with exon 1 of the *aspa* gene. The gene trap approach allowed to re-capitulate the activity of the endogenous aspa promoter by expression of the lacZ reporter in central and peripheral tissues. In fact, we found that in wildtype mice, aspa mRNA quantities in kidney, small intestine and sciatic nerve were higher than in brain indicating a role for NAA deacetylation in peripheral tissues. The loss of ASPA in the periphery appears to be well-tolerated in humans because the pathology in CD is restricted to the brain [6]. The failure to degrade NAA and/or other unknown substrates in ASPA expressing peripheral organs might have indirect toxic consequences for the CNS without inducing major histological abnormalities in the periphery. While a previous study employed *in situ* hybridization and reported on the lack of aspa mRNA signals in sciatic nerve [17], we provide strong evidence for the presence of high levels of aspa mRNA in Schwann cells. NAA was reported in sciatic nerve and ASPA-dependent deacetylation might contribute to lipid synthesis during PNS myelination similar to the function of ASPA in oligodendrocytes [18], [19]. This raises the possibility that ASPA-deficiency might have detrimental effects for peripheral nerve integrity. However, *aspa^lacZ/lacZ^* sciatic nerve tissue showed no gross abnormalities (our unpublished results) confirming a report on the normal PNS of the spontaneous ASPA-null tremor rat [20]. Of the few reports on peripheral nerve tissue of CD patients, only one case showed axonopathy and demyelination, but no vacuolisation [21], suggesting that this unique peripheral lesion might represent a coincidence rather than a consequence of ASPA-deficiency. How can the remarkably abundant expression of ASPA in normal Schwann cells be reconciled with the grossly normal peripheral nerves of ASPA-deficient mice? The physiology of peripheral nerve fibres differs from those in the CNS and Schwann cells might be more resistant to the effects of ASPA-deficiency (i.e. decreased pools of acetate, NAA toxicity) than oligodendrocytes. Alternatively, there might be subtle functional abnormalities in the ASPA-null PNS that are yet to be identified.

Ultrahigh field proton magnetic resonance spectroscopy revealed a substantial increase in levels of NAA but also taurine and myo-inositol in thalamus, and to a lesser extent in prefrontal cortex of *aspa^lacZ/lacZ^* mutants. Increased taurine in the *aspa^lacZ/lacZ^* brain may contribute to preservation of neuronal function via GABA_A_-mediated hyperpolarisation of thalamic relay neurons [22]. The profound increase of NAA in the CD brain is unique among neurological conditions and NAA-toxicity had been proposed as a reason for neuronal dysfunction [23], [24]. The efflux of NAA from neurons to the extracellular space is accompanied by a co-transport of water molecules, causing the interstitial pressure to rise [25]. Myo-inositol is a major osmolyte and serves as the precursor to phosphatidylinositol, a major membrane phospholipid. Extracellular tonicity induces the transport of myo-inositol from the outside to the inside of neurons and astrocytes [26]. In the ASPA-deficient brain, the dramatic increase in astrocyte numbers and concomitant increase in interstitial pressure may lead to glial uptake of myo-inositol to prevent cytotoxic damage to hypertonic neurons. The contribution of myo-inositol to the metabolic pathology in CD patients might have been underestimated since studies using *in vivo* imaging of CD brains detected no or only a moderate increase in prefrontal cortex or cerebellar white matter, respectively [27], [28]. These human data did not provide information on the thalamus. Our results also showed only a slight increase in prefrontal cortex myo-inositol. However, the changes in thalamus were severe, indicating that more dramatic metabolic changes might occur in the thalamus of the CD brain.

Although the NAAG signal was not determined, down-regulation of the NAAG-degrading enzyme folh1 in mutants indicates that levels of this metabolite may be increased. NAAG is neuroprotective in a number of neurological diseases by inhibiting the release of glutamate and GABA after activation of presynaptic mGluR3 [29]. In fact, we found both neurotransmitters to be decreased in the *aspa^lacZ/lacZ^* brain indicating that this neuroprotective mechanism might be impaired. ASPA is not expressed in astrocytes, yet this cell type appears to be particularly vulnerable for the osmotic downstream effects of accumulated NAA because astroglia are integral for the metabolism of NAAG, NAA and myo-inositol.

Swellings and vacuolisation appear within astrocytes before they become more prominent in the interstitium of CD patients [16]. These abnormalities precede widespread neuronal damage and neurological deterioration suggesting that astroglia may buffer some of the excess metabolites to compensate extracellular tonicity, sustain axonal integrity and promote neuronal survival. Bergmann glia contained osmiophilic inclusions reminiscent of accumulations of glycogen granules. The identity of these electron-dense particles could not be determined and might represent advanced stages of intracellular digestion of phagocytosed material, or signs of direct damage to the Bergmann glia fibres.

Damaged CNS neurons have been reported in CD, the Nur7 mutant, the tremor rat and the *aspa^lacZ^* mouse [10], [30], [31], suggesting that changes in axon integrity and metabolic status of the neuron contribute to the complex pathology. ASPA expression has been detected in wildtype neurons [3], and one could speculate that ASPA-mediated degradation of NAA might become important in energetically challenged neurons [32]. This concept is supported by reports on increased *aspa* gene activity in peripheral tissues in non-CNS diseases and is yet to be shown to occur in neurons [13], [33]. The distinct anatomical regions where spongy degeneration and hypomyelination occurred might be accounted for by the fact that different types of oligodendrocytes show regional variation in their connectivity to astrocytes via gap junctions, an important factor in regulating osmotic and metabolic homeostasis [34].

Congenital, infantile and juvenile forms of CD showing variable disease onset and severity have been suggested, with the moderate infantile category being the most frequent form by far [6], [35]. The pathology observed in *aspa^lacZ/lacZ^* mutants includes region specific spongy vacuolisation of the CNS, elevated NAA levels, demyelination, gliosis, and impaired motor behavior. As such, this mouse line is an accurate model of infantile CD. The *aspa^lacZ/lacZ^* line has been derived from C57BL/6J ES cells and shows a considerably milder phenotype compared with the previously described Aspa-KO mouse [9]. The latter line was obtained after the targeted deletion of the *aspa* gene in SV129 ES cells and shows motor seizures and premature death occurring as early as at two months of age. It is tempting to speculate that the genetic background can modify disease severity. Another major difference between the two lines is that the *aspa^lacZ^* allele was targeted with a promoterless selection marker while in Aspa-KO mice the *neo* gene is driven by the strong *pgk* promoter that has been suggested to interfere with the activity of genes adjacent to *aspa* [10], [36]. Consequently, the severe phenotype of the Aspa-KO mouse was suggested to be caused by multiple gene effects [10] that might also account for subtle differences i.e. unchanged folh1 activity in Aspa-KO mice [37] versus 50% folh1 mRNA reduction in *aspa^lacZ/lacZ^* mice (this study). Interestingly, homozygous *aspa^Nur7^* transgenic mice, carrying an *aspa* nonsense mutation identified in an ENU mutagenesis screen [10], show virtually the same mild neurological and histological abnormalities as the targeted *aspa^lacZ/lacZ^* line. Additional ENU-induced mutations in other genes of the Nur7 mutant cannot be excluded, but both *aspa^lacZ^* and Nur7 mutants are on the C57BL/6J background eliminating genetic strain differences. In summary, the *aspa^lacZ^* mouse is unique because the underlying genetic modification is restricted exclusively to the *aspa* gene, does not influence adjacent genes, and allows for screening of *aspa* gene activity.

Behavioural phenotyping of *aspa^lacZ^* mutants revealed sex-specific consequences of ASPA-deficiency. Rotarod performances were equally impaired in female and male mutants while different aspects of locomotor behaviour and inherent anxiety as monitored in the open field arena revealed sexual dimorphisms. There are no reports on gender-specific differences in behavioural test performance for the ASPA-KO or Nur7 mouse lines. However, a recent study using the natural ASPA-null *tremor* rat [8] showed that locomotor activity is less impaired in female *tremor* rats than males [38]. What mechanisms could account for these observations? Sexual dimorphism of oligodendrocytes in rodents and humans has been described and there is evidence for androgens to control oligodendrocyte differentiation and survival by signaling through their receptors expressed by oligodendroglia (reviewed in [39]). Therefore, the vulnerability of ASPA-deficient oligodenroglia may be different between female and male *aspa^lacZ/lacZ^* mutants and this could translate into differences at the behavioural level.

In summary, *aspa^lacZ/lacZ^* mice represent the accurate model of Canavan disease, and this line, as well as conditional *aspa^f/f^* mutants derived from this line, will be essential research tools to genetically dissect the mechanisms underlying CD and to design and test effective treatment strategies.

## Materials and Methods

### Ethics statement

Experiments were approved by the local animal care committee (Landesuntersuchungsamt Koblenz, permit number 23177/G10-1-036).

### Generation of aspa^lacZ^ mutant mice

Using homologous recombination in C57bl/6 embryonic stem (ES) cells, the βgeo cassette (including the genes encoding β-Galactosidase and Neomycin) was introduced into intron 1 of the aspa gene by the European Mouse Mutagenesis Consortium (EUCOMM). In addition, the critical exon 2 was flanked by loxP sites. An automated high-throughput approach and genotyping by long-range PCR (for technical see http://www.eucomm.org/) led to the identification of two independent aspa-lacZ C57bl/6 ES cell clones (H09 and C09) showing homologous targeting. We injected both clones into balb/c blastocysts and chimeric males were bred to C57Bl/6J females to produce germ line transmission. The C09-derived line was used for further experiments. We intercrossed heterozygous (*aspa^lacZ/+^*) offspring to obtain homozygous mutant mice (*aspa^lacZ/lacZ^*) and correct targeting was confirmed by Southern blot analysis. DNA was digested with ApaI and SmaI (NEB, Ipswich, MA), separated on a 0.7% agarose gel, blotted onto Hybond N+ membrane (GE Healthcare, Dassel, Germany) and probed with a 600 bp ^32^P-labelled fragment of the Neo-cassette. Mice were genotyped by PCR using Primer 1 as forward primer and Primer 3 as reversed primer resulting in a 336 bp band for the wild-type allele and a 387 bp band for the synthetic allele. Presence of the loxP site was detected by PCR with Primer 1 as forward primer and Primer 2 as reversed primer yielding a 307 bp band. Primer sequences are available upon request. *aspa^f/f^* mice were generated via flp-mediated recombination and deletion of the ßgeo cassette after crossing *aspa^lacZ/+^* animals with flp-deleter mice [40].

### RNA isolation and Q-PCR

Animals were sacrificed at postnatal day P60, organs dissected quickly, and snap frozen. Brains were homogenized under liquid nitrogen using mortar and pestle. Total RNA was isolated using the Nucleo-Spin RNAII-Kit (Macherey-Nagel, Dueren, Germany). 450 ng of DNase-treated total RNA was reverse-transcribed using High Capacity cDNA Reverse Transcription Kit (Applied Biosystems, Carlsbad, CA). The cDNA equivalent to 22.5 ng RNA was amplified using commercial TaqMan assays (Applied Biosystems, Carlsbad, CA) for mouse aspartoacylase (aspa; Mm00480867_m1), glucoronidase β (gusb; Mm00446953_m1) and folate hydrolase (folh1; Mm00489655_m1) with an ABI 7300 real time PCR cycler (Applied Biosystems, Carlsbad, CA). Quantitative RT-PCR reactions were performed in triplicates. Data analysis was done using a relative expression software tool (REST) using gusb as the reference gene [41].

### Antibody generation

Expression of recombinant human aspartoacylase fused to a C-terminal 6xHis tag using the bacterial expression vector pET-3a (New England Biolabs, Beverly, MA) was expressed in E. Coli (BL21) as described [2]. After induction with IPTG (1 µM) for 4 h, bacteria were sonicated in a solubilization buffer containing 20 mM Na-phosphate (pH 7.4), 0.5 M NaCl, 5 mM imidazole. Sonicated bacteria were pelleted, insoluble (pellet) and soluble (supernatant) fractions were separated by PAGE and ASPA content analyzed by coomassie staining. Less than 5% of total ASPA protein was located in the supernatant. Recombinant soluble ASPA was purifed by affinity chromatography using Ni-charged agarose (Ni-NTA Agarose, Quiagen, Hilden, Germany). On the day of injection, purified ASPA (0.1 mg) was mixed with Freund’s adjuvant and administered i.p. to a rabbit. Every two weeks the dose was repeated over a total of 12 weeks. Finally, the animal was sacrificed, blood collected and the serum isolated.

### Histology

For immunohistochemistry mice were deeply anesthetized with pentobarbital and trans-cardially perfused with phosphate bufferd saline (PBS), followed by phosphate buffered 4% paraformaldehyde. Brains were post-fixed in PFA over night and cryoprotected in 30% sucrose/PBS for 2 days, then cut into 40 µm free-floating sections using a Microm HM-560 cryostat (Thermo Scientific, Waltham, MA) as described [31] and stored at 4°C in cryoprotection solution (25% glycerin, 25% ethylene glycol and 50% PBS) until use. After washing with PBS, permeabilization with 0,2% TritonX-100 in PBS (PBS-Tx) and blocking in 4% normal goat serum (NGS) in PBS-Tx, sections were incubated overnight at 4°C with a combination of the following antibodies in 4% NGS in PBS-Tx: rabbit anti-aspa serum (1∶1000); chicken anti-β-Galactosidase (1∶500, abcam); mouse anti-GFAP (1∶1000, Sigma-Aldrich, St. Luis, MO), respectively. For Iba-1 and β-Galactosidase detection, antigen retrieval was performed prior to permeabilization by rinsing free floating sections two times in PBS followed by incubation in 10 mM sodium citrate buffer (pH 8.6) at 80°C for 30 min. Sections were allowed to cool down to room temperature in the same solution followed by permeabilization with PBS-Tx, blocking for 30 min in PBS-Tx 1% BSA (for anti-Iba-1) or NGS (for β-Gal), and incubation with the primary antibodies in blocking solution overnight at 4°C. Sections were washed with PBS and incubated with appropriate Alexa-conjugated secondary antibodies (1∶1000, Invitrogen, Carlsbad, CA) for 1–2 h at room temperature in 4% NGS in PBS-Tx, mounted on slides and coverslipped with Mowiol (Calbiochem, Darmstadt, Germany). For X-Gal histochemistry *aspa^lacZ/+^* animals were perfused in ice-cold 5% PFA, and tissues dissected and postfixed for 1 h followed by embedding in Kaiser’s gelatine (Merck, Darmstadt, Germany). Vibratome sections (60 µm; Microm, HM650 V; Thermo Scientific, Waltham, MA) were collected in PBS and incubated in X-gal solution (1.2 mg/ml X-gal, 5 mM potassium ferrocyanide and 2 mM MgCl_2_ in PBS) for 4–12 h. After 3 washes in PBS sections were mounted and coverslipped in Mowiol. For Nissl staining, sections were mounted onto glass slides in PBS and air-dried, then stained for 10 s with toluidine blue (0.1% in H_2_O). Slides were washed two times with H_2_O, destained for 15 s in 70% ethanol / 0.001% acetic acid, dehydrated in 100% ethanol and air-dried. After dipping in xylol, sections were mounted with Histokit (Carl Roth, Karlsruhe, Germany). Immunostaining was visualized using a Leica DMRA inverted microscope (Leica Microsystems, Wetzlar, Germany) or a Zeiss LSM 710 confocal microscope (Carl Zeiss MicroImaging, Jena, Germany). Teased sciatic nerve preparations were done as described [42]. Briefly, peripheral nerves were fixed by perfusion or immersion in 4% PFA in 0,1 M phosphatebuffer, pH 7,4 for 30–60 min. After several washes with phosphate buffer, nerves were teased on SuperFrostPlus slides in a 50 µl drop containing PBS, and air-dried. Primary antibodies were anti ASPA and rat anti MBP (1∶1000; Abcam).

### Electron microscopy

Animals were anesthetized with Avertin and perfused transcardially with warm HBSS followed by fixing solution (4% formaldehyde and 2.5% glutaraldehyde in phosphate buffer containing 0.5% NaCl as described [43]. The CNS was dissected and embedded in Epon (Serva) after postfixation with 2% OsO_4_ (Science Services, Munich, Germany) and dehydration with ethanol and isopropanol and propylenoxid. Ultrathin sections were cut using an Ultracut S ultramicrotome (Leica, Austria), mounted on 100 mesh hexagonal copper EM grids (Plano, Germany) and poststained with 4% aqueous uranyl acetate (SPI-Supplies, USA) and lead citrate [44]. The sections were analyzed in a LEO EM 912AB electron microscope (Zeiss, Oberkochen, Germany), and pictures were taken with an on-axis 2048×2048 CCD camera (Proscan, Scheuring, Germany).

### Immunoblotting

Animals were sacrificed at P60, organs dissected quickly and snap frozen. Brains were homogenized under liquid nitrogen using mortar and pestle. Aliquots were sonicated in tris buffered saline (TBS) containing protease inhibitors (Complete, Roche Applied Science, Mannheim, Germany) and protein concentration was determined by the method of Bradford. 20 µg of protein were mixed with 5x Laemmli reducing sample buffer, denatured for 5 min at 95°C (with exception of samples allocated for detection of PLP), separated by SDS-PAGE and transferred onto nitrocellulose membranes (Protran, Whatman; GE Healthcare, Dassel, Germany) Membranes were probed with the following antibodies: rabbit anti-aspa serum (1∶1000), mouse anti-tubulin (1∶400000, Sigma-Aldrich, St. Luis, MO), mouse anti-CNPase (1∶1000, Abcam, Cambridge, UK), rat anti-MBP (1∶1000; Abcam, Cambridge, UK), rat anti-PLP aa3 (1∶1000, gift of J. Trotter). Antibodies were detected by the appropriate HRP-conjugated secondary antibodies (Dianova, Hamburg, Germany) followed by ECL-detection.

### Behavioural testing

Differences in innate anxiety and general locomotor behavior of male and female *aspa^lacZ/lacZ^* (males: n = 8; females: n = 7) and *aspa^+/+^* (males: n = 8; females: n = 6) mice were assessed in the open field test at P90. Animals were placed in the center of an arena (40×40×40 cm) under bright light conditions (100 lux) and monitored for 30 min. Total distance traveled and time spent in the center area and in the periphery was analyzed using Smart software (Panlab, Madrid, Spain). Using the same cohorts of animals, motor learning was assessed using the rotarod test at P90. Mice were familiarized with the apparatus (Ugo Basile, Comerio, Italy) in two trials for two min at constant speed (4 rpm). In a series of 3 trials per animal, the time that the mice remained on the accelerating roller (4–40 rpm in 4 min) was scored. Between trials the animals were allowed a 10 min period in their home cage. The individual performances were averaged over 2 days.

### ^1^H MR spectroscopy

A male control mouse and an aspa-lacZ mouse (4 months) where measured with ^1^H-Magnetic resonance spectroscopy on a 9.4T Bruker BioSpec scanner (Bruker, Rheinstetten, Germany) equipped with a cryogenic very low temperature, closed cycle cooled RF-coil. Mice were anesthetized by a gas mixture of O_2_: 50% and air: 50% with ca. 1.8% isoflurane. Respiration rate was monitored throughout the experiment. Body temperature was maintained at 37°C by warm water circulation and verified by a rectal thermo sensor. For voxel positioning and high resolution images of the mouse brain were acquired with a T2-weighted RARE (rapid acquisition with relaxation enhancement) (TE = 11 ms, TR = 2.5 s, alpha  = 20°, two averages; Resolution 66*66*500 um^3^). Spectra were acquired using a PRESS single voxel Sequence at an echo time of TE = 10 ms from a 12 µl (2×2×3 mm^3^) volume from the thalamic region, with a repetition time of TR = 4 s and 256 averages (NEX) resulting in a total acquisition time of 17 min per spectrum (Fig. 7). Water suppression was done with the VAPOR (Variable pule powers and optimized relaxation delays) method interleaved with 6 outer-volume suppression slices. To ensure optimal B0 homogeneity 1st and 2nd order shimming was conducted with Fastmap over a volume of 5×5×5 mm^3^ prior each metabolite measurement resulting in a line width (fwhm) of the unsuppressed water signal of 12 Hz or better. An additional one shot unsuppressed waterline (RF-off) was acquired for each voxel, which was used for eddy-current correction and water referencing. Quantification was done according with LCModel by fitting the *in vivo* spectra to phantom data of 16 metabolite solutions measured at the same scanner. Macromolecule contributions were fitted through simulated spectra at the relevant frequencies. Concentration values were referenced to an unsuppressed water signal acquired from the same voxel assuming a mean water-concentration of 46.1 M/l.

### Statistics

All graphs and statistical analyses were done with GraphPad Prism 4 software (La Jolla, CA). Student’s t-test was used for statistical analysis. Myelin protein levels were analysed with One-Way ANOVA followed by Bonferroni post-hoc test. Values are presented as the mean ± s.e.m and *p* <0.05 was considered as statistically significant.

## Supporting Information

Figure S1 **β-Galactosidase co-localizes with ASPA.** Laser-confocal immunodetection of β-Galactosidase (A) and ASPA (B) in the thalamus of an *aspa^lacZ/+^* reporter mouse. (C) The nuclear DAPI staining shows all cells in the tissue. (D) The merged picture shows β-Galactosidase immunoreactivity in ASPA-positive oligodendrocytes (arrowheads) but not in other cells (asterisks). Bar: 20 µm.
(TIF)

Figure S2 **EM analysis of optic nerve.** There are no obvious histological differences between the control (A) and mutant (B) optic nerve. Bar: 500 nm.
(TIF)

Figure S3 **Downregulation of folh1 expression in the absence of ASPA.** Q-PCR analysis for detection of folh1 in total RNA isolated from whole brains of *aspa^+/+^*, *aspa^lacZ/+^* and *aspa^lacZ/lacZ^* littermates (P60, n = 3) shows decreased folh1 mRNA in homozygous mutants. *aspa^+/+^* expression levels were used as a nominator.
(TIF)

Video S1 **Gait abnormalities of** ***aspa^lacZ/lacZ^*** **mice.** Side-by-side comparison of representative motor behaviour in an open field. The split screen shows a male control mouse (left side in horizontal split, top in vertical split) and an *aspa^lacZ/lacZ^* littermate (right side in horizontal split, bottom in vertical split) at two months of age.
(WMV)
