# Supplementary figures and images for: Aspartoacylase-LacZ Knockin Mice: An Engineered Model of Canavan Disease
Source: PLoS One. 2011 May 20;6(5):e20336. doi: 10.1371/journal.pone.0020336 (PMC3098885; doi:10.1371/journal.pone.0020336)

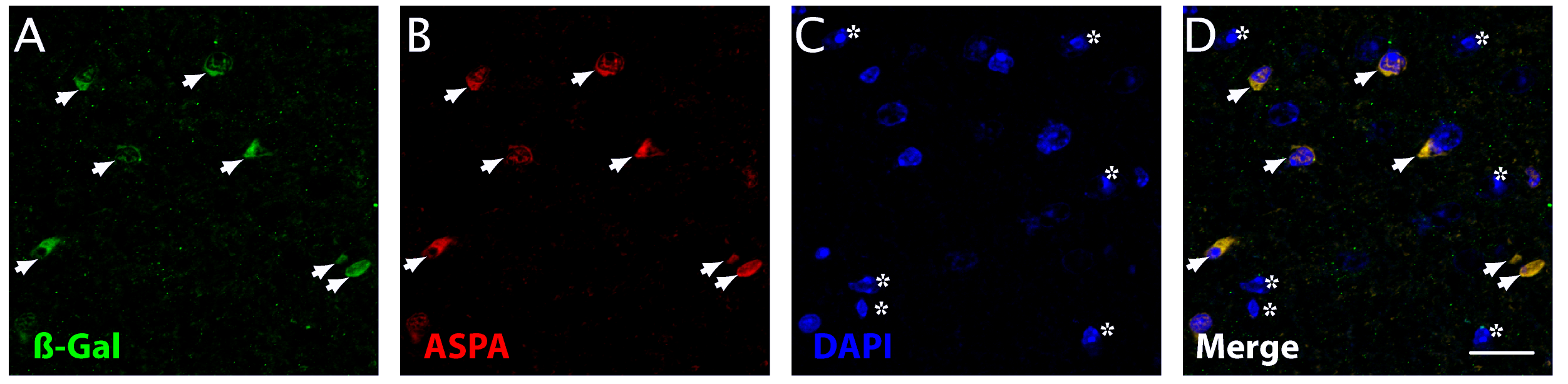

Supplement: Figure S1 — β-Galactosidase co-localizes with ASPA. Laser-confocal immunodetection of β-Galactosidase (A) and ASPA (B) in the thalamus of an aspalacZ/+ reporter mouse. (C) The nuclear DAPI staining shows all cells in the tissue. (D) The merged picture shows β-Galactosidase immunoreactivity in ASPA-positive oligodendrocytes (arrowheads) but not in other cells (asterisks). Bar: 20 µm. (TIF) [file pone.0020336.s001.tif]

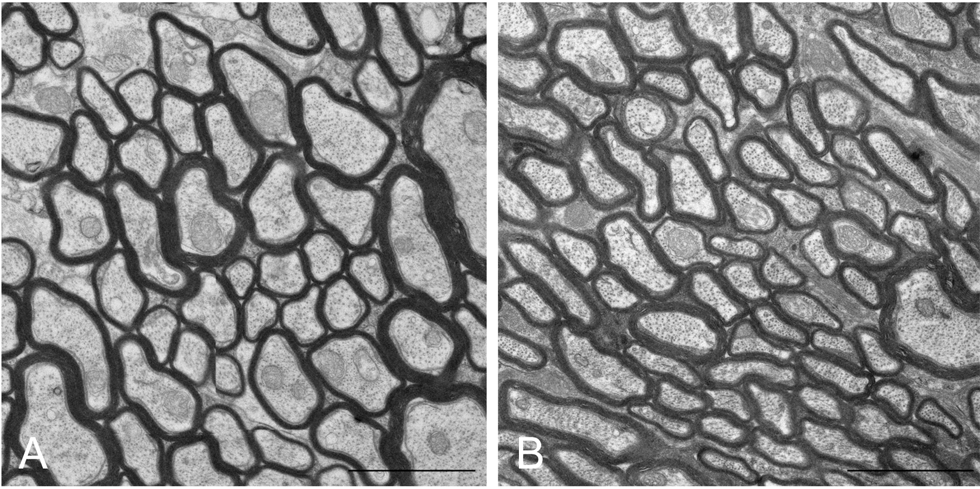

Supplement: Figure S2 — EM analysis of optic nerve. There are no obvious histological differences between the control (A) and mutant (B) optic nerve. Bar: 500 nm. (TIF) [file pone.0020336.s002.tif]

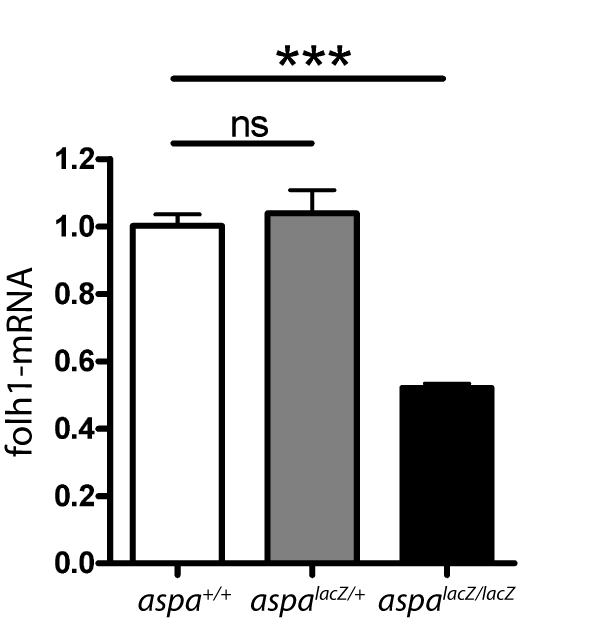

Supplement: Figure S3 — Downregulation of folh1 expression in the absence of ASPA. Q-PCR analysis for detection of folh1 in total RNA isolated from whole brains of aspa+/+, aspalacZ/+ and aspalacZ/lacZ littermates (P60, n = 3) shows decreased folh1 mRNA in homozygous mutants. aspa+/+ expression levels were used as a nominator. (TIF) [file pone.0020336.s003.tif]
